# Impact of MELD Allocation System on Waiting List and Early Post-Liver Transplant Mortality

**DOI:** 10.1371/journal.pone.0155822

**Published:** 2016-06-14

**Authors:** Juan Jurado-García, María Muñoz García-Borruel, Manuel Luis Rodríguez-Perálvarez, Patricia Ruíz-Cuesta, Antonio Poyato-González, Pilar Barrera-Baena, Enrique Fraga-Rivas, Guadalupe Costán-Rodero, Javier Briceño-Delgado, José Luis Montero-Álvarez, Manuel de la Mata-García

**Affiliations:** 1 Department of Hepatology and Liver Transplantation. Reina Sofía University Hospital, Córdoba, Spain; 2 IMIBIC, Maimonides Biomedical Research Institute, Córdoba, Spain; 3 CIBERehd (Networked Biomedical Research Center in Hepatic and Digestive Disease); 4 Department of Hepatobiliary Surgery and Liver Transplantation. Reina Sofía University Hospital. Córdoba, Spain; University of Navarra School of Medicine and Center for Applied Medical Research (CIMA), SPAIN

## Abstract

**Background and aims:**

MELD allocation system has changed the clinical consequences on waiting list (WL) for LT, but its impact on mortality has been seldom studied. We aimed to assess the ability of MELD and other prognostic scores to predict mortality after LT.

**Methods:**

301 consecutive patients enlisted for LT were included, and prioritized within WL by using the MELD-score according to: hepatic insufficiency (HI), refractory ascites (RA) and hepatocellular carcinoma (HCC). The analysis was performed to predict early mortality after LT (8 weeks).

**Results:**

Patients were enlisted as HI (44.9%), RA (19.3%) and HCC (35.9%). The major aetiologies of liver disease were HCV (45.5%). Ninety-four patients (31.3%) were excluded from WL, with no differences among the three groups (p = 0.23). The remaining 207 patients (68.7%) underwent LT, being HI the most frequent indication (42.5%). HI patients had the shortest length within WL (113.6 days vs 215.8 and 308.9 respectively; p<0.001), but the highest early post-LT mortality rates (18.2% vs 6.8% and 6.7% respectively; p<0.001). The independent predictors of early post-LT mortality in the HI group were higher bilirubin (OR = 1.08; p = 0.038), increased iMELD (OR = 1.06; p = 0.046) and non-alcoholic cirrhosis (OR = 4.13; p = 0.017). Among the prognostic scores the iMELD had the best predictive accuracy (AUC = 0.66), which was strengthened in non-alcoholic cirrhosis (AUC = 0.77).

**Conclusion:**

Patients enlisted due to HI had the highest early post-LT mortality rates despite of the shortest length within WL. The iMELD had the best accuracy to predict early post-LT mortality in patients with HI, and thus it may benefit the WL management.

## Introduction

The imbalance between donors and candidates for liver transplantation (LT) provides the rationale for the creation of waiting lists. The allocation based on time on waiting list (WL) was too simplistic and increased waiting list mortality in the past. In order to minimize WL-mortality several systems have been developed to prioritize patients based in principles of justice (“sickest first”), equality and efficiency. The Model for End-stage Liver Disease (MELD) was first described as a prognostic tool to predict early mortality in patients with cirrhosis undergoing TIPS **[1],** and it was further validated in patients awaiting LT, in whom the MELD score was strongly associated with short-term survival **[2]**. The MELD score is based in three objective serum parameters (ie. bilirubin, creatinine and international normalized ratio-INR) combined according the following equation: 9.57 x [Ln creatinine (mg/dL)] + 3.78 x [Ln bilirubin (mg/dL)] + 11.2 x Ln INR] + 0.643 **[1].**

The main advantage of the MELD score is that it contains objective parameters able to be revised whenever necessary. However some groups of patients are not adequately prioritized by the MELD score either because the risk of death is not directly related to the liver function (but to complications of portal hypertension; ie: refractory ascites), or because the progression of the underlying liver disease is the adverse outcome (rather than death; ie. hepatocellular carcinoma). Most institutions apply an empirical correction of the MELD score in these so called “MELD exceptions” in order to balance the access to LT between groups. Recent studies suggested that the empirical correction of MELD may impair the access to LT of those patients who accessed the waiting list with the classic MELD allocation system **[3]**. Several modifications of the original MELD formula have been developed to correct this imbalance **[4] [5]** but they are not routinely implemented in clinical practice.

Otherwise, as MELD-based allocation has reduced significantly the WL-mortality by improving the organ access for the sickest patients, other consequences have been observed: mean-MELD score at the moment of transplantation has increased and higher early post-transplant mortality has been reported by several authors **[6].** Donors graft quality has worsened in recent years, and suboptimal donors provide up to 50% of organs **[7].** These factors may impact on post-operative mortality, which is a concern for most of LT institutions. The identification of risk factors of WL and post-LT mortality is mandatory.

A critical concept when evaluating a LT program is the intention to treat analysis: the results should be evaluated not only in terms of mortality within the WL and drop-out rates, but also considering the outcomes after LT. An ideal prioritization system would be able not only to allocate an organ to the patient with the highest risk of drop-out, but also with an expected positive outcome after LT. Hitherto none of the tested allocation systems have demonstrated any predictive ability for post-LT outcomes, particularly for early death after LT.

The aims of the present study were: a) to assess the predictive capacity of the MELD and other indexes to predict mortality in WL and early mortality post-LT, and b) to analyze whether the underlying liver disease modifies this predictive capacity.

## Methods

This is an observational retrospective analysis of a prospectively collected database in which 301 consecutive patients enlisted for LT between 2008 and 2013 were included. Patients under 18 years old, retransplanted or transplanted with acute liver failure were excluded. Follow up lasted until drop out, for those patients excluded from WL, or until 8 weeks after LT (for transplanted patients). In each patient the following variables were collected: Demographic data, underlying liver disease, comorbidities, reason for drop-out, length within waiting list, pre-LT routine blood tests, surgery features and transfusion need, early post-LT complications, immunosuppression, hospital stay, donors characteristics and histology of the liver graft (steatosis and ischemia reperfusion injury). The prognostic scores were calculated immediately before LT (ie. MELD, MELD-Na, iMELD, uMELD y UKELD).

The present study was conducted in Córdoba, one of the four LT institutions available in Andalucía (Spain) with a reference population of 8.4 million. Patients were included in the waiting list according to one of the following groups: hepatic insufficiency (HI), refractory ascites (RA) or hepatocellular carcinoma (HCC). In patients with hepatic insufficiency the MELD score was updated according to changes in blood tests. In patients included under refractory ascites (with 15 points), one extra MELD point was added every three months up to 18 points, and 1 extra MELD points every other month thereafter (from the first month using the MELD-Na). Patients enlisted for HCC, extra MELD points were added to the base of 15 points only in the subgroup considered at increased risk of tumour progression beyond Milan criteria (ie. a single tumour ≥3cm, multinodular or with alpha fetoprotein>200 ng/mL). In such patients one extra-MELD point was added every month within waiting list **[8].** The patients with HCC on WL were treated by radiofrequency ablation or trans-arterial chemoembolization whenever necessary. Apart from the MELD score **[1],** several modifications of the MELD were also calculated by using the originally described formulas. Some of these were designed for a more accurate prediction of outcome in patients with cirrhosis: sodium corrected MELD (MELD-Na **[9]**), United Kingdom End-Stage Liver Disease, (UKELD **[10]),** Integrated MELD which includes age and serum sodium (iMELD **[11])**. Others had a different weighting of their components in the model such as the Update-MELD (uMELD **[12]**). The calculation of each prognostic score was performed as follows: **MELD-Na score [6]:** MELD + (140-Na [mmol/L])– 0.025 x MELD x (140-Na [mmol/L]). **iMELD [8]:** MELD + age (years) x 0.3–0.7 x Na (mmol/L) + 100. **UKELD [7]:** 5.395 x ln (INR) + 1.485 x ln (creatinine [μmol/L]) + 3.13 x ln (Bilirubin [μmol/L])– 81.565 x ln (Na [mmol/L]) + 435. **uMELD score [9]:** 1.266 x ln (1 + creatinine [mg/dl]) + 0.94 x ln (1 + (Bilirubin [mg/dl]) + 1.658 x ln (1 + INR).

### Statistical analysis

Statistical analysis was performed by using SPSS 22.0 (IBM, Chicago, USA). Variables were displayed in frequency tables or expressed as means and standard deviations, except for those with asymmetric distribution in which median and interquartile range were used. Chi square test was used for frequencies, student’s T or ANOVA tests for quantitative variables, and Mann-Whitney’s U or Kruskal-Wallis for asymmetric distributions. A multiple logistic regression was designed to identify the independent predictors of early mortality after LT (ie. 8 weeks), and to control for possible confounding factors. Those variables with p<0.30 in the univariate analysis were selected to enter the initial model. The elimination of not significant variables was done in a backward stepwise process. All possible interactions between the included variables were tested and considered significant if their combination in the model reached a p<0.05. The predictive ability of each prognostic score was calculated by using ROC curves. Every hypothesis was two-tailed and considered significant if p<0.05.

The present study was designed and performed according to the ethical principles contained in the declaration of Helsinky. Patients included in the study provided written informed consent and the database was de-identified prior to analysis to keep anonymity. The project was approved by the Andalusian public health **S**ystem **E**thic's **C**ommittee (SEC).

## Results

**Fig 1:**
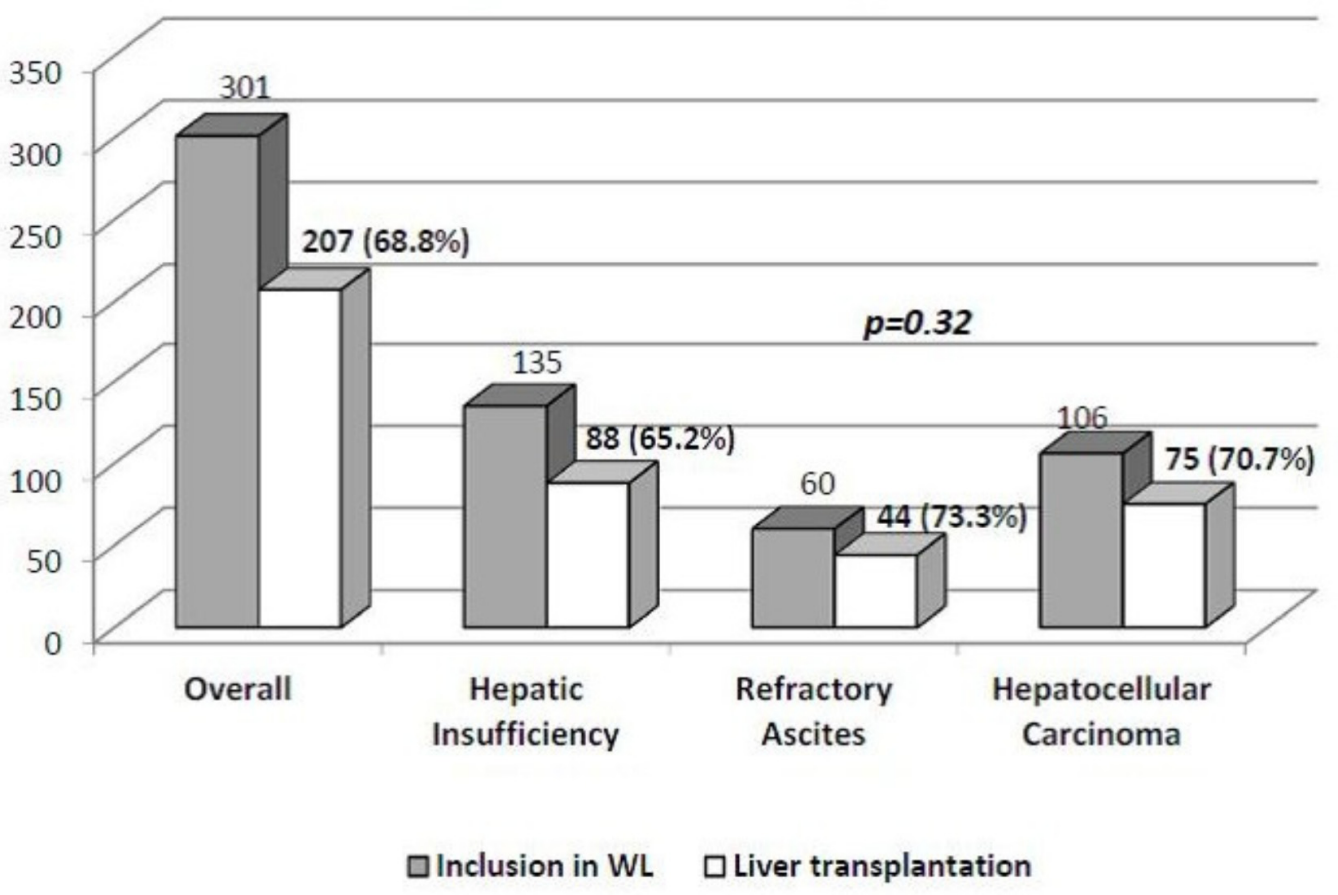
Probability of liver transplantation

Among 301 patients included 240 were men (79.7%) with a mean age of 54.5 ± 7.6 years (the baseline characteristics of the study group are presented in Ta**ble 1**). The reason for inclusion in WL was hepatic insufficiency in 44.8% (n = 135) of patients, refractory ascites in 19.9% (n = 60) and hepatocellular carcinoma in 35.3% (n = 106). The most frequent underlying liver disease was chronic hepatitis C (n = 137, 45.5%) followed by alcoholic liver disease (n = 110, 36.5%). The main features of the included patients and the probability of being transplanted depending on the inclusion criteria for LT are presented in **Fig 1**.

**Table 1:** Main baseline characteristics of the study group.

**Age** (mean± SD) (range)	54.6 ± 7.7 (31–73)				
**Gender** (male/female, %)	240/61 (79.7/20.3)				
	**Hepatic insufficiency**	**Refractory ascites**	**Hepatocellular Carcinoma**	**Overall**	
**Indication for LT**(n, %)	135 (44.8)	60 (19.9)	106 (35.3)	301	
**Liver transplantation** (n, %)	88 (42.5)	44 (21.3)	75 (36.2)	207 (68.8)	
**Probability of LT**	65.2%	73.3%	70.7%	68.8%	p = 0.32
**Exclusion rates on WL Reason for drop-out (n, %)**	47 (34.8)	16 (26.7)	31 (29.2)	94 (31.2)	p = 0.23
**Global mortality on WL** (n, %)	21 (15.6)	9 (15.0)	6 (5.7)	36 (11.9)	P<0.001
**Early mortality rates post-LT**(n, %)	16 (18.2)	3 (6.8)	5 (6.7)	24 (11.6)	p<0.001
	9 (13.8%)	——	——	——	——
**MELD < 24 (n = 65)**	7 (30.4%)				
**MELD ≥ 24 (n = 23)**					

Determinants of early mortality post-LT (MELD ≥ 24)

**Bilirubin** (mg/dl): 18.1 vs 9.5; p = 0.02

**Hospital stay post-ICU** (days): 4.7 vs 14.7; p = 0.022

**Fig 2:**
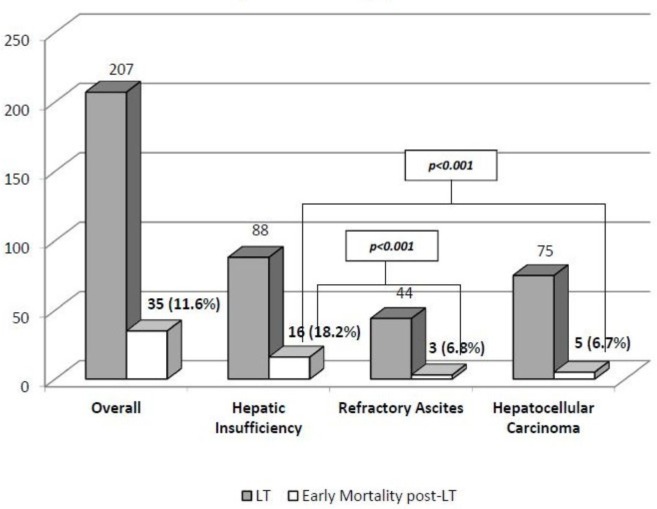
Exclusion rates of WL

Ninety-four patients (31.2%) were excluded from the waiting list because improved liver function (26.6%), progression of the hepatocellular carcinoma beyond Milan criteria (20.2%), mortality (includes serious deterioration of liver function) (38.3%) or other causes (14.9%) such as patient refusal (n = 2), concomitant malignancies (n = 2), cardiopulmonary and cerebrovascular disease (n = 6), psychiatric disorders (n = 1) or active addiction (n = 1). Exclusion rates depending on the inclusion criteria for LT were: 34.8% (47/135) for patients with HI, 26.7% (16/60) for patients with RA, and 29.2% (31/106) for patients with HCC, with no significant differences among groups (p = 0.23). Overall mortality rate within waiting list was 11.9%. Mortality within WL was the most frequent cause of drop-out in patients with hepatic insufficiency and refractory ascites (n = 21 (15.6%) and n = 9 (15.0%), respectively), whereas tumour progression was the most frequent reason for drop-out in patients with HCC (n = 19; 17.9%) (**See Fig 2**). The risk factors of mortality within the WL among patients with hepatic insufficiency were increased MELD score (p<0.001), as well as other derived prognostic scores such as iMELD (p = 0.041), uMELD (p = 0.003) and UKELD (p<0.001). Serum bilirubin and INR were also increased in patients with HI who died within waiting list (p = 0.006 and p = 0.011 respectively). In the group with refractory ascites the mortality was positively correlated with the MELD score (p = 0.004), MELD-Na (p = 0.004), iMELD (p = 0.0012), UKELD (p = 0.005), uMELD (p = 0.024) and sodium (p = 0.042). **Table 2.**

**Table 2:** Determinants of mortality on the waiting list: hepatic insufficiency group and refractory ascites group.

	HI (death vs alive)	p	RA (death vs alive)	p
**Bilirubin (mg/dl)**	11.4 vs 4.4	*0*.*006*	—-	
**INR**	1.9 vs 1.5	*0*.*011*	—-	
**Sodio (mmol/L)**	—-		133.5 vs 138.9	*0*.*042*
**MELD**	21.3 vs 17.5	*<0*.*001*	17.5 vs 11.3	*0*.*004*
**MELD-Na**	—-		18.5 vs 12.4	*0*.*004*
**iMELD**	42.8 vs 38.1	*0*.*041*	40.1 vs 31.6	*0*.*012*
**UKELD**	60.7 vs 55.6	*0*.*001*	55.7 vs 50.4	*0*.*024*
**uMELD**	4.7 vs 3.8	*0*.*003*	3.5 vs 3.1	*0*.*04*

**Fig 3:**
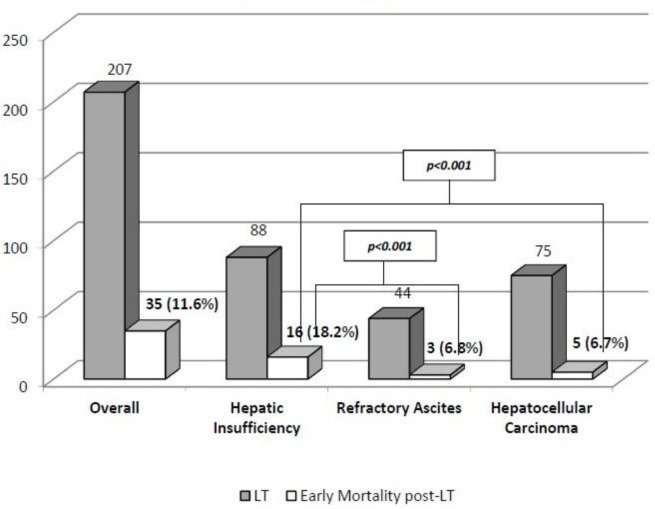
Early mortality post-LT

The remaining cohort underwent LT (n = 207; 68.7%). The median stay on waiting list reached 191.9 days (range: 2–776). The most frequent criterion to be included in waiting list in the LT cohort was hepatic insufficiency (n = 135, 44.8%), followed by hepatocellular carcinoma (n = 106, 35.3%) and refractory ascites (n = 60, 19.9%). Among patients with HI, the underlying liver disease was hepatitis C in 42.1% (n = 87) of patients and alcoholic liver disease in 37.7% (n = 78) of patients. The uncorrected pre-LT MELD score was 20.3 ± 5.6 in patients with HI, 14.2 ± 5.2 (MELD-Na: 19.6 ± 4.7) in RA group, and 11.2 ± 4.2 in HCC group. The subgroup of hepatic insufficiency had the shortest waiting length for LT (113.6 ± 14.9 days), when compared with patients with hepatocellular carcinoma (215.8 ± 15.3 days) and refractory ascites (308.9 ± 36.6 days) (p<0.001 in both comparisons). The early overall post-LT mortality reached 11.6% (n = 24) and was higher for patients transplanted for hepatic insufficiency (18.2%, n = 16) if compared with refractory ascites (6.8%, n = 3) and hepatocellular carcinoma (6.7%, n = 5) with significant differences (p = 0.044 and p = 0.020, respectively). **See Fig 3.** Early mortality post-LT was especially high in patients with hepatic insufficiency and MELD score ≥ 24. Serum bilirubin pre-LT and length in hospital stay post- ICU (Intensive Care Unit) were determinants of mortality.

The baseline characteristics of the LT patients with hepatic insufficiency compared with those from the remaining groups are presented in **Table 3**. There were significant differences in terms of ascites (p = 0.005), liver function tests (INR, p = 0.001 and PT, p = 0.005) and the different prognostic scores evaluated (MELD (p = 0.001), iMELD (p = 0.001), uMELD (p = 0.001), y UKELD (p = 0.001)) between patients with hepatic insufficiency and refractory ascites. When comparing the group with HI and HCC these differences are even more sound and pertains to pre-LT complications of cirrhosis (ie. ascites, p = 0.001, hepatic encephalopathy, p = 0.001, variceal bleeding, p = 0.019 and renal failure, p = 0.019), serum tests (INR, bilirubin, sodium, albúmin; p<0.001 and creatinine, p = 0.025) and prognostic scores (MELD, iMELD, uMELD and UKELD, p<0.001). There were no differences in terms of the surgery approach or in the histology features of the graft liver (grade of steatosis and ischemia reperfusion injury).

**Table 3:** Baseline characteristics of transplanted patients: hepatic insufficiency compared with refractory ascites and HCC groups.

		RA	*p*	HI	*p*	HCC
**n** (%): 207		44 (21.3)		88 (42.5)		75 (36.2)
**Age** (years ± SD): 54.5 ± 7.6		54.5 ± 8.3	*NS*	53.8 ± 8.3	*0*.*001*	55.45 ± 7.1
**Sex** (M/F, %): 79.2 /20.8		70.5/29.5	*NS*	75.0/25.0	*0*.*018*	89.3/10.7
**Comorbidities** (%)	Arterial hypertension	6.8	*NS*	13.6	*0*.*014*	29.3
	DiabetesDiabetes	27.3	*NS*	18.2	*0*.*010*	36.0
	Respiratory disease	4.5	*NS*	9.1	*NS*	9.3
	Neurologic disease	2.3	*NS*	2.3	*NS*	5.3
	Cardiovasc. disease	6.86.8	*NS*	9.1	*NS*	13.3
	Obesity	4.5	*NS*	9.1	*NS*	10.7
**Aetiology of liver disease** (%)	Alcohol	40.9	*NS*	37.7	*0*.*045*	27.9
	HCV	45.7	*NS*	42.1	*0*.*02*	65.3
	Others	13.4	*NS*	20.3	*NS*	6.8
**Hepatic decompensation pre-LT** (%)	Ascites	100.0	*0*.*005*	84.1	*0*.*001*	29.3
	Hep. encephalophaty	55.8	*NS*	60.9	*0*.*001*	13.3
	Gastroint. bleeding	205.0	*NS*	28.4	*0*.*019*	13.3
	S. bacterial peritonitis	18.2	*NS*	9.1	*NS*	2.7
	Renal failure	20.5	*NS*	31.8	*0*.*019*	16.0
**Stay on waiting list** (days)	Mean: 191.9; Median: 166; Range: 2–776; IQR: 59–256	308.9 ± 36.6	*0*.*001*	113.6 ± 14.9	*0*.*001*	215.8 ± 15.3
**Hospital stay pre-LT** (days ± DS)		1.1 ± 1.2	*0*.*001*	3.8 ± 7.9	*0*.*001*	0.7 ± 0.9
**Transfusion requirement** (units)	Erythrocytes	6.2 ± 4.8	*NS*	6.95 ± 6.50	*0*.*012*	4.48 ± 5.16
	Platelets	0.8 ± 0.9	*NS*	1.06 ± 1.11	*NS*	0.97 ± 1.30
	Plasma	3.5 ± 3.1	*NS*	4.55 ± 4.01	*0*.*007*	2.93 ± 3.07
**Surgery** (hours)	Ischemia	6.8 ± 2.2	*NS*	6.4 ± 2.3	*NS*	6.1 ± 2.3
	Surgery	4.3 ± 0.7	*NS*	4.3 ± 1.3	*NS*	4.4 ± 1.3
**Histology of liver graft** (%) (M/Mo/S*)	Steatosis	39.5/55.8/4.7	*NS*	44.6/42.2/13.3	*NS*	61.3/28/10.7
	Preservation	44.2/48.8/7.0	*NS*	59.5/34.5/6.0	*NS*	55.4/32.4/12.2
**Post-LT complications**(%)	Acute reject	27.7	*NS*	27.7	*NS*	22.7
	Renal failure	56.8	*NS*	65.1	*0*.*028*	45.3
	Neurological	40.9	*NS*	47.0	*NS*	30.7
	Infection	33.2	*NS*	37.8	*NS*	30.7
	Cardiovascular	18.2	*NS*	20.5	*NS*	12.0
	Arterial thrombosis	7.2	*NS*	7.2	*NS*	8.0
**Pre-LT blood test**	Bilirubin (mg/dl)	2.9 ±1.9	*NS*	7.1 ± 6.1	*0*.*001*	2.1 ± 2.5
	Creatinine (mg/dl)	0.9 ± 0.3	*NS*	1.1 ± 0.8	*0*.*025*	0.9 ± 0.3
	Sodium (mmol/l)	133.2 ± 6.3	*0*.*001*	133.4 ± 7.1	*0*.*001*	137.3 ± 4.4
	INR	1.3 ± 0.2	*0*.*005*	1.7 ± 0.4	*0*.*001*	1.2 ± 0.2
	Prothrombin time (s)	17.5 ± 3.9	*NS*	19.9 ± 4.9	*0*.*001*	14.9 ± 2.6
	Albumin (gr/dl)	3.1 ± 0.8	*NS*	2.9 ± 0.7	*0*.*001*	3.8 ± 0.7
**Prognostic scores**	**MELD**	14.2 ± 5.2	*0*.*001*	20.3 ± 5.6	*0*.*001*	11.2 ± 4.2
	**iMELD**	36.9 ± 6.7	*0*.*001*	42.6 ± 8.7	*0*.*001*	32.3 ± 6.1
	**uMELD**	3.4 ± 0.6	*0*.*001*	4.3 ± 0.7	*0*.*001*	3.1 ± 0.6
	**UKELD**	55.4 ± 5.1	*0*.*001*	59.3 ± 6.4	*0*.*001*	51.3 ± 4.3

In the univariate analysis the early mortality in transplanted patients with HI was related to non-alcoholic aetiology (p = 0.012), higher serum bilirubin (p = 0.024), transfusion need (erythrocytes (p = 0.002) and plasma (p = 0.027)), pre-LT hospital stay (p = 0.007) and increased iMELD (p = 0.039). However the pre-LT MELD score was not associated with post-LT early mortality (p = 0.26). In the multivariate analysis the independent predictors of early mortality after LT were: non-alcoholic aetiology (OR = 4.13; 95% CI: 1.29–13.23; p = 0.017), serum bilirubin (OR = 1.08; 95%CI: 1.005–1.176: p = 0.038) and iMELD (OR = 1.064; 95%CI: 1.001–1.132; p = 0.046) (**Table 4**).

**Table 4:** Factors related with early post-LT mortality in hepatic insufficiency group.

	Univariate analysis		Multivariate analysis		
	Death (Yes vs No)	*p*	OR	95% CI	*p*
**Non-alcoholic aetiology**	68.8% vs 31.3%	*0*.*012*	4.136	1.29–13.23	*0*.*017*
**Transfusion requirement** (units)	**Erythrocytes**: 14.8 ± 6.8 vs 6.3 ±0.5 **Plasma**: 8 ± 5.5 vs 4.2 ± 3.7	*0*.*002 0*.*027*	—	—	*—*
**Bilirubin** (mg/dl)	10.3 ± 9.9 vs 6.4 ± 4.8	*0*.*024*	1.087	1.01–1.18	*0*.*038*
**iMELD**	46.6 ± 9.7 vs 41.7 ± 8.2	*0*.*039*	1.064	1.01–1.13	*0*.*046*

**Fig 4:**
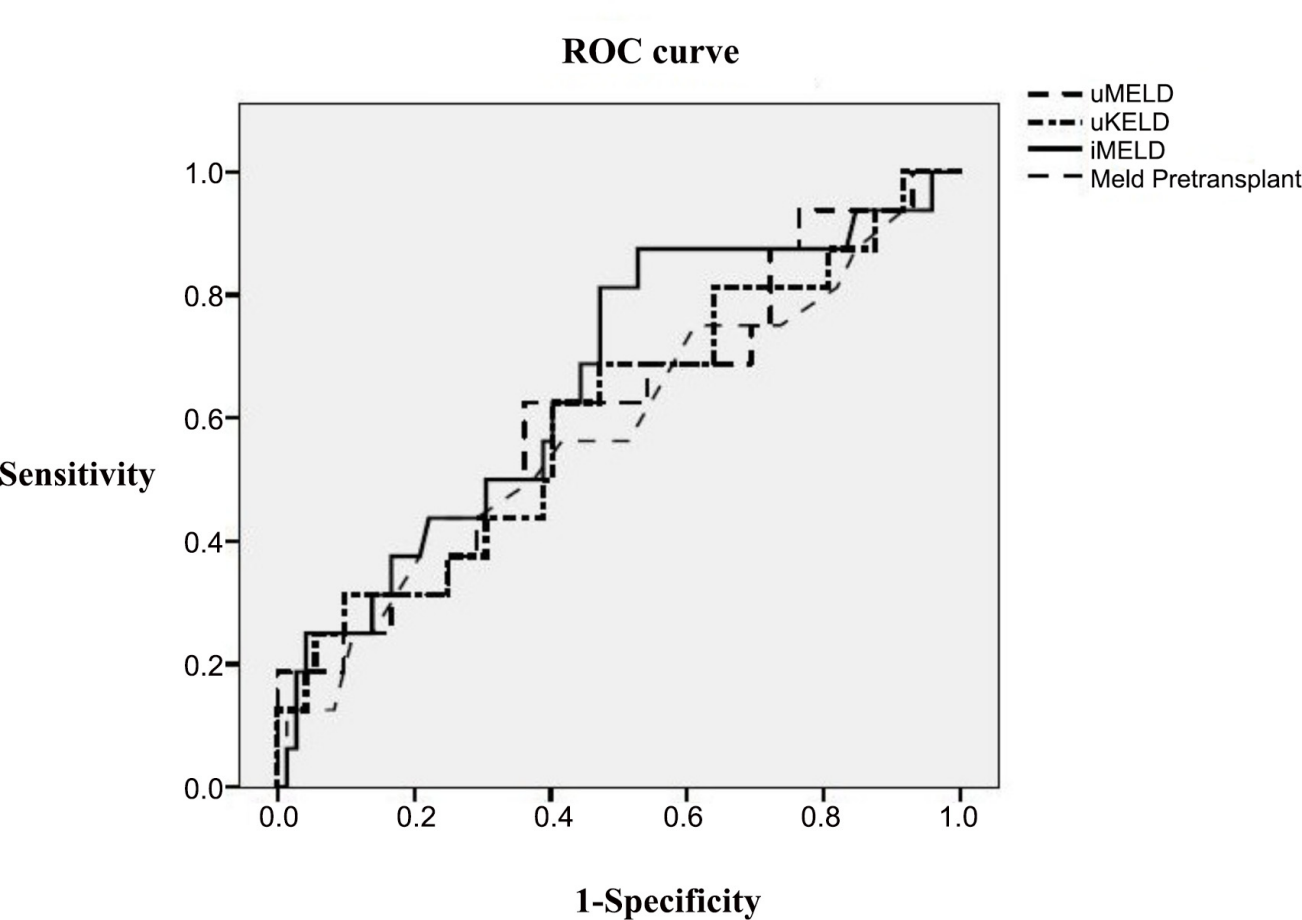
ROC curves. Comparison of all prognostic scores. Predictive ability of early post-LT mortality in hepatic insufficiency group.

**Fig 5:**
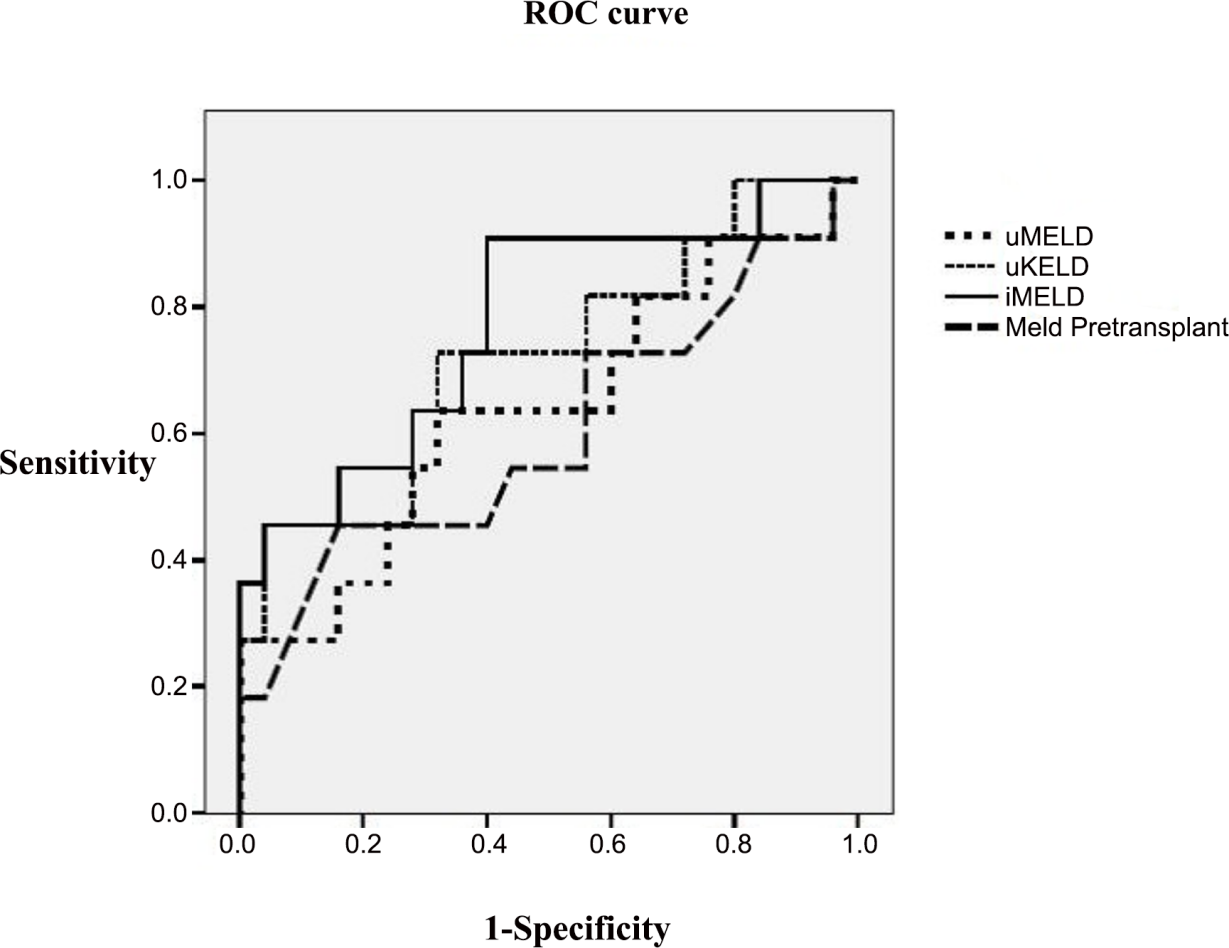
Predictive ability of all prognostic scores in transplanted patients (hepatic insufficiency and non alcoholic aetiology group).

The ROC curves of the evaluated prognostic scores in the whole LT population are presented in the **Fig 4**. The predictive ability of the iMELD score was increased (AUROC = 0.664), although with no significant differences from the remaining scores, and with suboptimal accuracy. However the predictive accuracy of the iMELD increased significantly in the subgroup of patients transplanted with HI without alcoholic liver disease (AUROC = 0.775) (**Fig 5**), particularly when serum bilirubin was >10 mg/dl (AUROC = 0.950); although the limited number of patients (n = 12) does not allow to generalize the results in this subgroup.

In patients with refractory ascites the risk of early mortality after LT was related with serum sodium (p = 0.03) and surgery blood transfusion need (erythrocytes, p = 0.001, platelets, p = 0.012 and plasma, p = 0.006 respectively), but neither of these parameters, nor any of the evaluated prognostic scores, reached statistical significance in the multivariate logistic regression.

## Discussion

LT is the standard of care to treat patients with end stage liver disease or with hepatocellular carcinoma within Milan criteria. The imbalance between the increasing number of candidates for LT and the limited and unchanged pool of donors makes it critical to establish a prioritization system that ensures an optimized allocation policy. The MELD score provides accurate information about short term mortality within the waiting list, but its relationship with post-LT outcomes is a matter of debate, as recently shown in a systematic review of 37 studies **[13].** The addition of new parameters to the MELD formula resulted in an increased accuracy to predict mortality within the waiting list **[4, 14],** but unfortunately the ability to predict post-LT outcomes remained unchanged.

There are many clinical situations in which the risk of adverse outcomes within the waiting list is not related to a worsening of the liver function, and thus the MELD score is not useful. These are the so called MELD exceptions, among which the refractory ascites and the HCC are the most frequent. In a recent study an increased probability of being transplanted was described for patients included in waiting list as MELD exceptions, particularly for patients with hepatocellular carcinoma (72.4% for hepatocellular carcinoma, 70.8% for other MELD exceptions, and 44.6% for patients with hepatic insufficiency)**[3].** The imbalance to access LT among groups of indication was translated into doubled mortality rates within the WL for patients included with HI (26.1%), when compared with patients with HCC (10.2%) or with other MELD exceptions (11.3%). In the present study the access to LT was balanced between groups and there were similar rates of drop-out in patients with hepatic insufficiency as in the remaining cohort, suggesting that the prioritization system used provided an adequate allocation policy. In patients with HI the MELD score, as well as the other derived prognostic scores evaluated, were closely associated with the mortality rates within the WL, in line with previously published studies **[1, 9–12].** However in the group included with refractory ascites only those prognostic scores which included the serum sodium reflected somehow the risk of adverse outcomes within the waiting list, although this relationship was not confirmed in the multivariate analysis, probably because of the reduced number of patients composing this subgroup.

The MELD score measures the hepatic and renal function. Patients included with hepatic insufficiency are expected to show a higher MELD score as a consequence of a severe impairment of the liver function. In patients with refractory ascites or hepatocellular carcinoma a worsening of their clinical situation is not usually accompanied by an increase in the MELD score. Thus an empirical correction of the MELD score is needed to balance the access for LT between groups. In the present study the access to LT was balanced between groups as stated above, and patients with HI had the shortest length within the WL, suggesting that this group would be favored by the currently used prioritization system. However the early mortality after LT was increased in patients with hepatic insufficiency, especially in patients with MELD≥24, when compared with the remaining cohort, and these adverse outcomes were not related with the MELD score. The iMELD behaved as a more accurate predictor of post-LT outcomes than the conventional MELD score, particularly for patients with non alcoholic aetiology and very impaired liver function (serum bilirubin >10 mg/dL). In previous studies the iMELD, which incorporates also age and serum sodium, has been proposed as a more accurate predictor of mortality within the waiting list than the conventional MELD score **[12],** but its relationship with post-LT outcomes has not been evaluated so far. Indeed the serum sodium mirrors the haemodynamic dysfunction which is a major source of complications **[14–15]**, and age is a well know prognostic factor in the cirrhotic population **[16–17]**. According to our results the implementation of the iMELD as the prioritization system for patients with non-alcoholic liver disease, particularly if they have severely impaired liver function, would benefit significantly the waiting list management and would provide a more rational prioritization, allowing for an improved post-LT outcomes in these difficult to manage patients.

In patients with hepatocellular carcinoma the addition of extra-MELD points in an empirical fashion extremely reduced the risk of drop-out due to tumor progression beyond Milan criteria, but also resulted in an excessive prioritization of these patients as compared with other LT indications in earlier experiences **[5].** Nowadays only those patients with HCC at increased risk of tumour progression beyond Milan criteria (ie. a single tumour ≥3cm, multinodular or with alpha fetoprotein>200 ng/mL) receive extra-MELD points as the time within the WL passes **[18].** Some authors have proposed new models to correct this imbalance and to reflect more accurately the risk of tumor progression within the WL **[19–22].** In contrast to a recent study, in which the candidates with hepatocellular carcinoma had a shorter length within the WL and increased transplantation rates **[23],** our current system of empirical correction of MELD in patients with HCC balanced drop-out rates between groups. It is highly possible that an extra prioritization of patients with hepatocellular carcinoma would reduce the drop-out rates in this group, and would avoid tumour progression in some patients. However in absence of a significant increase in the number of donors, this strategy would surely boost the rates of pre and post-LT mortality in patients with hepatic insufficiency. In addition it has been suggested in a large population-based study that a significant shortening in the waiting length for LT in patients with hepatocellular carcinoma would increase the rates of tumour recurrence after LT, because patients with highly aggressive tumours, who would have been excluded thereafter because of rapid progression, would undergo LT **[24].**

As reported in patients with hepatocellular carcinoma, the group of refractory ascites had a prolonged length within the waiting list when compared with patients with hepatic insufficiency, but drop-out rates were reduced. Hyponatremia is frequent in patients with end-stage liver disease and ascites, and it is a well known biomarker of haemodynamic impairment and mortality within the waiting list and LT mortality **[25].** Conventional MELD score does not mirror the clinical severity in these patients, and the implementation of serum sodium to the formula (MELD-Na) provided more accurate prognostic information **[26]**. On the other hand the serum sodium is highly variable and may be influenced by many external factors such as dehydratation or diuretic therapy, thus preventing for the systematic use of MELD-Na, UKELD and iMELD in most LT institutions. In our study the serum sodium was equally reduced in patients with refractory ascites as in the remaining cohort (135.1 ± 4.9 vs 135.3 ± 4.7; p = 0.74). In spite of this some of the scores which implemented serum sodium (ie. UKELD, iMELD) were able to predict the risk of drop-out univariately (but not multivariately) in patients with RA. The obtained results suggest that our prioritization system provides similar outcomes in patients with refractory ascites when compared with other indications for LT, despite of the increased length in WL.

The limitations of the present study are its retrospective design, the single-centre recruitment and the limited sample size. However the data were obtained from a prospectively collected database in which the outcomes are updated in a monthly fashion, thus ensuring the reliability of the analyses performed and the results obtained. Furthermore an intention-to-treat analysis was performed, which is central in the LT setting, although seldom attempted in previous studies. A broader analysis including more institutions may be warranted, but the quality of the data would be more difficult to assess. It is noteworthy that the prognostic scores evaluated were calculated immediately prior to LT. Some previous studies have reported a benefit when the ΔMELD within the waiting list is used **[27],** but this strategy is not routinely implemented in most LT institutions, and neither it is in our centre.

In conclusion, our currently used allocation system prioritizes the most those patients with hepatic insufficiency but there is still an imbalance in terms of post-LT outcomes, according to which these patients have increased early mortality rates after LT. The iMELD score behaved as a more rational approach to prioritize the subgroup of patients with hepatic insufficiency, being particularly useful to discriminate among patients with non-alcoholic aetiology and increased conventional MELD score. Further studies are needed to validate these results in a wider LT population.

## Abbreviations

AUC: Area under curve
HI: Hepatic insufficiency
INR: International normalized ratio
ICU: Intensive Unit Care
HCC: Hepatocellular carcinoma
HCV: Hepatitis chronic C
iMELD: Integrated MELD
LT: Liver transplantation
MELD: Model for End-Stage Liver Disease
RA: Refractory ascites
RFA: Radiofrequency ablation
TACE: Transarterial chemoembolization
uMELD: Update MELD
UKELD: United Kingdom End-Stage Liver Disease
WL: Waiting list
